# Mobility and associations with levels of cerebrospinal fluid amyloid β and tau in a memory clinic cohort

**DOI:** 10.3389/fnagi.2023.1101306

**Published:** 2023-02-06

**Authors:** Gro Gujord Tangen, Karen Sverdrup, Kristin Taraldsen, Karin Persson, Knut Engedal, Peter Bekkhus-Wetterberg, Anne-Brita Knapskog

**Affiliations:** ^1^The Norwegian National Centre for Ageing and Health, Vestfold Hospital Trust, Tønsberg, Norway; ^2^Department of Geriatric Medicine, Oslo University Hospital, Oslo, Norway; ^3^Department of Rehabilitation Science and Health Technology, Oslo Metropolitan University, Oslo, Norway

**Keywords:** gait, balance, mobility, dual task, amyloid, tau, biomarker, Alzheimer

## Abstract

**Background:**

Mobility impairments, in terms of gait and balance, are common in persons with dementia. To explore this relationship further, we examined the associations between mobility and cerebrospinal fluid (CSF) core biomarkers for Alzheimer’s disease (AD).

**Methods:**

In this cross-sectional study, we included 64 participants [two with subjective cognitive decline (SCD), 13 with mild cognitive impairment (MCI) and 49 with dementia] from a memory clinic. Mobility was examined using gait speed, Mini-Balance Evaluation Systems test (Mini-BESTest), Timed Up and Go (TUG), and TUG dual-task cost (TUG DTC). The CSF biomarkers included were amyloid-β 42 (Aβ_42_), total-tau (t-tau), and phospho tau (p-tau_181_). Associations between mobility and biomarkers were analyzed through correlations and multiple linear regression analyses adjusted for (1) age, sex, and comorbidity, and (2) SCD/MCI vs. dementia.

**Results:**

Aβ_42_ was significantly correlated with each of the mobility outcomes. In the adjusted multiple regression analyses, Aβ_42_ was significantly associated with Mini-BESTest and TUG in the fully adjusted model and with TUG DTC in step 1 of the adjusted model (adjusting for age, sex, and comorbidity). T-tau was only associated with TUG DTC in step 1 of the adjusted model. P-tau_181_ was not associated with any of the mobility outcomes in any of the analyses.

**Conclusion:**

Better performance on mobility outcomes were associated with higher levels of CSF Aβ_42_. The association was strongest between Aβ_42_ and Mini-BESTest, suggesting that dynamic balance might be closely related with AD-specific pathology.

## 1. Introduction

Dementia is by WHO declared a public health priority (World Health Organization, 2012; World Health Organization, 2017). Globally, it is estimated that there were 57.4 million persons living with dementia in 2019, and this number is projected to increase to 152 million in 2050 (Nichols et al., 2022). Alzheimer’s disease (AD) is the most common cause of dementia, accounting for 57% (GjOra et al., 2021) to 80% (Alzheimer’s Association, 2022a) of all dementia cases. Although there is no curative treatment to date, a timely and accurate diagnosis is important for ruling out potential reversible conditions, provision of information and counseling, facilitating access to services and optimizing care, support of lifestyle changes that may delay progression, and possibility of participation in clinical trials (Liss et al., 2021).

Traditionally, a clinical diagnosis of dementia and AD is based on thorough anamnestic interviews with the patient and an informant, cognitive tests, assessment of neurological, physical, and psychiatric symptoms, supported by brain imaging (Medbøen et al., 2022). However, neuropathological changes occur up to decades before clinical manifestation of cognitive and functional impairments (Jack and Holtzman, 2013). With the current perspective of AD and other dementias as conditions with long-term pathophysiological changes occurring in clinical asymptomatic individuals, the use of biomarkers representing underlying pathology has been established for diagnostic purposes. The first detectable pathology of AD in the cerebrospinal fluid (CSF) is reduced concentration of amyloid-β 42 (Aβ_42_), followed by an increase in phosphorylated tau (P-tau) and total-tau (T-tau; Palmqvist et al., 2019). Lower concentration of Aβ_42_ in CSF represents abnormal aggregation of Aβ deposits in the brain, whereas increased P-tau signifies formation of neurofibrillary tangles in the neuron. These are considered highly specific for AD, while increased T-tau is a marker for neurodegeneration (Palmqvist et al., 2019). Although positron emission tomography (PET) imaging with tracers is now used to detect concentrations of Aβ and tau in the brain (Maschio and Ni, 2022), both PET and CSF biomarkers are unavailable in many settings (Frisoni et al., 2017). In a global perspective, there is an urgent need for simple tools that can help to improve the etiological of diagnosis of dementia.

Mobility, in terms of gait and balance, entails complex cognitive and motor processes, engaging several parts of the central nervous system (Valkanova and Ebmeier, 2017). Meta-analyses have concluded that poor gait performance predicts development of cognitive impairment and dementia (Beauchet et al., 2016; Sekhon et al., 2019). Other aspects of mobility, such as balance and dual-task cost, are also strongly associated with cognitive function, in particular with executive function (Muir et al., 2012; Tangen et al., 2014). The dual-task cost is the effect a cognitive task (e.g., talking or counting) has on gait (Montero-Odasso et al., 2009). To enhance the understanding of the specific nature of the relationship between mobility and cognitive decline, exploration of associations with underlying pathology is needed.

Studies have investigated the relationship between mobility and CSF AD core biomarkers in people with cognitive impairment and dementia. In a pilot study of 17 patients with mild AD, higher gait variability was associated with reduced Aβ_42_/Aβ_40_ and Aβ_42_/Aβ_38_ ratios (Koychev et al., 2018). In two larger studies of memory clinic patients, worse performance on dual-task gait tasks was associated with presence of tau-pathology, but not with Aβ-pathology (Ahman et al., 2019; Muurling et al., 2020). In the largest study to date (*n* = 299), including cognitively unimpaired older adults and memory clinic patients with subjective cognitive decline (SCD) or mild cognitive impairment (MCI), Nilsson et al. found that while a pathologic Aβ_42_/Aβ_40_ was associated with impaired balance and worse dual-task performance in the total sample, these associations were not statistically significant in the MCI group alone (*n* = 99; Nilsson et al., 2021). P-tau_181_ was associated with dual-task cost in the total sample (*n* = 240) as well as in the MCI group (Nilsson et al., 2021). So far, the results are not conclusive, and more studies are needed to elucidate such as which measure of mobility outcome is closest related to the CSF AD core biomarkers.

The overall aim of this study was to explore the associations between four different aspects of mobility and AD core biomarkers in CSF in memory clinic patients with all-cause MCI and dementia, as well as in a subsample with MCI and dementia due to AD.

## 2. Methods

### 2.1. Participants

This study combines two different cross-sectional cohorts from the memory clinic at Oslo University Hospital, Ullevål, Norway. Cohort 1 (*n* = 26) was included in a study conducted from January 2011 to August 2012, whereas cohort 2 (*n* = 38) was included in another study conducted from January 2017 to July 2019. All participants were included in The Norwegian registry of persons assessed for cognitive symptoms (NorCog; Medbøen et al., 2022). Most of them were included at their first visit to the memory clinic, but some were included at follow-up visits. All participants had to have a diagnosis of SCD, MCI, or dementia, be home-dwelling, and be able to walk independently without a walking device. In cohort 1, only dementia patients with AD were included. Patients with SCD and MCI in both cohorts and patients with dementia in cohort 2 were included regardless of etiology. We excluded participants who needed an interpreter to conduct the cognitive assessments and participants with moderate to severe degree of dementia, or severe hearing or vision impairments. Only participants where AD core biomarkers from CSF had been analyzed within 6 months before or after the mobility assessments were included.

### 2.2. Diagnostic procedures

All participants were examined following a comprehensive standardized research protocol including clinical interviews with the patients and their informants, a comprehensive cognitive test battery, physical examination, blood and CSF examination, and imaging (MRI or if not feasible CT; Medbøen et al., 2022). Based on all available information, the patients were classified according to the National Institute of Aging and the Alzheimer’s Association (NIA-AA)—core-criteria for MCI (Albert et al., 2011) and dementia (McKhann et al., 2011). Further, patients were subclassified for probable or possible AD or AD with mixed pathology according to etiological diagnoses using the NIA-AA-criteria. Dementia with Lewy bodies (DLB) was diagnosed using the revised criteria from the DLB Consortium (McKeith et al., 2017). For primary progressive aphasia (PPA), the recommendations from Gorno-Tempini et al. (2011) were used. From the cognitive test battery, we used the Mini-Mental State Examination (MMSE) to describe global cognition (Folstein et al., 1975).

### 2.3. CSF procedures

The CSF AD core biomarkers were analyzed at Department of Interdisciplinary Laboratory Medicine and Medical Biochemistry, Akershus University Hospital (AHUS) by enzyme-linked immunosorbent assays (ELISA; Innotest® hTau Ag, phoshoTau (181P) and β-amyloid 1–42 Fujirebio Europe, Gent, Belgium). For the present study, we used Aβ_42_, T-tau, and P-tau_181_ (Aβ_42/40_ is not available in Norway). The laboratory is part of the Alzheimer’s Association QC program for CSF biomarkers (Alzheimer’s Association, 2022b).

### 2.4. Ethics

All participants provided informed written consent to participate in the study. Participation was voluntary, and participants could withdraw from the study at any time. Both studies included in this paper were approved by the Regional Committee for Medical and Health Research Ethics South East Norway; ref. 2010/2363 (cohort 1) and ref. 2016/1119 (cohort 2) and the OUS’s Data Protection Officer. The ethics committee also approved the merging of the data of the two studies.

### 2.5. Mobility outcomes

Gait speed was obtained from a 6-meter walk test in cohort 1 and from a 4-meter walk test in the Short Physical Performance Battery (Guralnik et al., 1994) in cohort 2. Apart from the distance, the procedure was identical in both settings. From a standing position, the patients were asked to walk at their usual gait speed. The test was conducted twice and the best time of the two trials was used as the outcome (m/s).

The Mini-Balance Evaluation Systems Test (Mini-BESTest) was used as a measure of dynamic balance (Franchignoni et al., 2010). The Mini-BESTest consists of 14 items divided into 4 sections (anticipatory postural adjustments, postural responses, sensory orientation, and stability in gait). Each item is scored from 0 to 2, with a total score of maximum 28 (as the best score). The Mini-BESTest has been validated in neurological populations (Franchignoni et al., 2010) and the Norwegian version has also been validated (Hamre et al., 2017).

Timed Up and Go (TUG) was used as a measure of overall mobility. Participants were asked to rise from a chair, walk 3 m at their usual pace to a line on the floor, turn around, and walk back to the chair and sit down again (Podsiadlo and Richardson, 1991). We also used the TUG in combination with a cognitive task (random numbers) as a measure of dual-task cost (TUG DTC; Horak et al., 2009). Participants were instructed to list random numbers while completing TUG at their usual gait speed. There were no instructions regarding which task should be prioritized. TUG DTC was calculated as the relative time difference between TUG and TUG with a cognitive task (100*(TUG with a cognitive task—TUG time)/TUG time). A negative TUG DTC indicates that the participant took longer time to complete the TUG with a cognitive task. Both the gait speed tests and the TUG tests were recorded by use of a handheld stopwatch.

The participants were asked “Do you have any injury or condition, such as arthritis, hip prosthesis, or previous fractures that may affect your gait or balance?” This information is used as a dichotomous variable for comorbidity of relevance for mobility. All mobility assessments were conducted by the same physiotherapist (GGT).

### 2.6. Statistical analysis

Descriptive analyses were done using mean and standard deviation for variables with normal distribution, and median and interquartile range for skewed variables. Categorical variables were described with numbers and percentages. To compare the demographical and clinical characteristics of the groups with SCD/MCI and AD, we used Chi-square tests, *t*-tests, or Mann–Whitney *U*-test when appropriate.

To examine the associations between mobility and CSF biomarkers, we first performed correlational analyses between each of the mobility outcomes and each biomarker, using Spearman’s rank correlation since some variables had a non-parametric distribution. We then proceeded with multiple linear regression analyses in two steps, with the four mobility outcomes (gait speed, Mini-BESTest, TUG, and TUG DTC) as dependent variables. We performed separate models for Aβ_42,_ T-tau, and P-tau_181_, and all models were adjusted for age, sex, and comorbidity (yes/no) in the first step, and then also for diagnostic status (SCD/MCI vs. dementia) in the second step. Independent variables were checked for issues regarding multicollinearity by performing correlation analyses (*r*_s_ *<* 0.7). Further, we inspected the residual plots of the regression models to check that we did not violate model assumptions. As sensitivity analyses, we conducted the same analyses in the subsample (*n* = 54) limited to MCI due to AD (*n* = 8) and AD dementia (*n* = 46). All statistical analyses were conducted in SPSS version 28, all tests were two-tailed, and the level of significance was set at *p <* 0.05.

## 3. Results

Descriptive data for the total sample are presented in Table 1, as well as comparisons according to severity of cognitive impairment. Only two patients had SCD; therefore, we have treated SCD and MCI (*n* = 13) as one group. The dementia group consisted of 46 patients with AD or mixed AD, two patients with DLB, and one patient with PPA. The dementia group had worse performance on the Mini-BESTest (*p* = 0.021) and worse TUG DTC (*p* < 0.001) than the SCD/MCI group.

**Table 1:** Characteristics of participants, according to severity of cognitive impairment.

	Total sample *N* = 64	SCD (*n* = 2) & MCI (*n* = 13)	Dementia *N* = 49	*p*
Age in years	68.8 (7.1)	67.7 (6.3)	69.1 (7.4)	0.53^a^
Men, *n* (%)	35 (54.7%)	8 (53.3%)	27 (55.1%)	1.0^b^
MMSE, median (IQR)	27.0 (7)	29.0 (1)	25.0 (6)	<0.001^c^
Comorbidity relevant for mobility, *n* (%)	14 (21.9%)	5 (33.3%)	9 (18.4%)	0.29^b^
Dementia etiology, *n*				<0.001^b^
AD/AD mixed		7 (46.7%)	46 (93.9%)	
Other		8 (53.3%)	3 (6.1%)	
Aβ_42_, pg./mL	686.0 (250.0)	881.6 (306.6)	626.1 (197.5)	<0.001^a^
P-tau_181_, pg./mL	81.9 (32.7)	69.9 (33.0)	85.5 (32.0)	0.10^a^
T-tau, pg./mL,	607.5 (300.6)	438.4 (297.9)	659.3 (284.7)	0.012^a^
Gait speed (m/s)	1.02 (0.3)	1.04 (0.16)	1.01 (0.19)	0.62^a^
Mini-BESTest, points	22.7 (3.6)	24.7 (3.0)	22.2 (3.5)	0.021^a^
TUG, s	10.8 (2.6)	9.9 (2.2)	11.1 (2.7)	0.14^a^
TUG DTC (%), median (IQR)	−16.3 (35.6)	−0.7 (14.8)	−21.8 (33.4)	<0.001^c^

Data is represented as Mean (SD), unless specified otherwise. MCI, mild cognitive impairment; Aβ_42_, amyloid-β 42; T-tau, total tau; MMSE, Mini-Mental State Examination; Mini-BESTest, Mini-Balance Evaluation Systems Test (0–28 points, higher score indicates better performance); TUG, Timed Up-and-Go; TUG DTC, Timed Up-and-Go dual-task cost.

a *t*-test.

b Chi-square test.

c Mann–Whitney *U*-test.

There were no significant differences between the two cohorts regarding demographic characteristics, MMSE, or CSF biomarkers (Table 2). However, cohort 1 had significantly higher gait speed than cohort 2 (1.08 vs. 0.98 m/s, *p* = 0.020), and worse TUG DTC performance (−31.5% vs. −15.5%, *p* = 0.035).

**Table 2:** Comparisons of participants’ characteristics in cohort 1 and cohort 2.

	Cohort1, *n* = 26	Cohort 2, *N* = 38	*p*
Age in years	69.4 (7.2)	68.3 (7.1)	0.57^a^
Men, *n* (%)	16 (61.5)	19 (50.0)	0.45^b^
MMSE, median (IQR)	27.0 (6)	27.0 (8)	0.89^c^
SCD & MCI, *n* (%)	4 (15.4)	11 (28.9)	0.25^b^
Aβ_42_, pg./mL	684.3 (259.3)	687.1 (247.0)	0.97^a^
P-tau_181_, pg./mL	80.5 (32.4)	82.8 (33.2)	0.78^a^
T-tau, pg./mL,	657.4 (332.0)	573.4 (276.5)	0.28^a^
Gait speed (m/s)	1.08 (0.2)	0.98 (0.2)	0.02^a^
Mini-BESTest, points	21.9 (3.1)	23.4 (3.8)	0.10^a^
TUG, s	11.1 (2.8)	10.6 (2.5)	0.49^a^
TUG DTC (%), median (IQR)	−28.3 (30.5)	−9.7 (24.8)	0.04^c^

Data is represented as Mean (SD), unless specified otherwise. MCI, mild cognitive impairment; Aβ_42_, amyloid-β 42; T-tau, total tau; MMSE, Mini-Mental State Examination; Mini-BESTest, Mini-Balance Evaluation Systems Test (0–28 points, higher score indicates better performance); TUG, Timed Up-and-Go; TUG DTC, Timed Up-and-Go dual-task cost.

a *t*-test.

b Chi-square test.

c Mann–Whitney *U*-test.

Aβ_42_ was significantly correlated with each of the mobility outcomes, with Spearman’s rank correlation coefficients ranging from 0.28 (gait speed) to 0.39 (Mini-BESTest). T-tau was significantly associated with TUG DTC (*r_s_ −*0.37; Table 3).

**Table 3:** Spearman’s rank correlation coefficients (*r*_s_) between mobility outcomes and CSF AD core biomarkers (*n* = 64).

Mobility outcome	Aβ_42_	P-tau	Total-tau
	*r*_s_, *p*	*r*_s_, *p*	*r*_s_, *p*
Gait speed	0.28, 0.024	−0.13, 0.31	−0.09, 0.46
Mini-BESTest	0.39, 0.002	0.01, 0.94	−0.09, 0.46
TUG	−0.35, 0.004	0.10, 0.44	0.18, 0.15
TUG DTC	0.37, 0.003	−0.22, 0.08	−0.37, 0.007

Mini-BESTest, Mini-Balance Evaluation Systems Test; TUG, Timed Up-and-Go; TUG DTC, Timed Up-and-Go dual-task cost.

Results from the adjusted multiple regression analyses are presented in Table 4. Aβ_42_ was significantly associated with Mini-BESTest (0.004 [0.002, 0.008], *p* = 0.040) and TUG (−0.003 [−0.006, <−0.001], *p* = 0.041) in the fully adjusted model and with TUG DTC (0.038 [0.009, 0.067], *p* = 0.012) in step 1 of the adjusted model (adjusting for age, sex, and comorbidity). T-tau was only associated with TUG DTC (−0.032 [−0.057, −0.006], *p* = 0.016) in step 1 of the adjusted model. P-tau_181_ was not associated with any of the mobility outcomes in any of the analyses. The adjusted explained variance in the regression models with significant associations ranged from 0.06 for the model with Aβ_42_ and TUG to 0.26 for the model with Aβ_42_ and Mini-BESTest.

**Table 4:** Associations between CSF AD core biomarkers and (i) Gait speed (m/s), (ii) Mini-BESTest, (iii) TUG, and (iv) TUG DTC (*n* = 64).

	A. Models adjusted for age, sex and comorbidity		B. Models adjusted for age, sex comorbidity and diagnostic group^b^	
	**(i) Gait speed**		**(i) Gait speed**	
	*B**^a^* (95% CI)	*p*	*B**^a^* (95% CI)	*p*
1. Aβ_42_	<0.0001 (<−0.0001, 0.0003)	0.11	0.0002 (<−0.0001, 0.0003)	0.139
2. P-tau	−0.001 (−0.002,0.0004)	0.145	−0.001 (−0.002,0.0004)	0.174
3. T-tau	<−0.0001 (−0.0002, 0.0001)	0.268	<−0.0001 (−0.0002, 0.0001)	0.333
	**(ii) Mini-BESTest**		**(ii) Mini-BESTest**	
1. Aβ_42_	0.005 (0.002, 0.008)	0.003	0.004 (0.0002, 0.008)	0.040
2. P-tau	−0.009 (−0.37, 0.019)	0.521	0.002 (−0.27, 0.31)	0.896
3. T-tau	−0.002 (−0.005, 0.001)	0.107	−0.001 (−0.004, 0.002)	0.481
	**(iii) TUG**		**(iii) TUG**	
1. Aβ_42_	−0.003 (−0.006, −0.001)	0.012	−0.003 (−0.006, −0.0001)	0.041
2. P-tau	0.012 (−0.009, 0.034)	0.249	0.009 (−0.012, 0.031)	0.385
3. T-tau	0.002 (<−0.0004, 0.004)	0.098	0.002 (−0.001, 0.004)	0.303
	**(iv) TUG DTC**		**(iv) TUG DTC**	
1. Aβ_42_	0.038 (0.009, 0.067)	0.012	0.024 (−0.007, 0.056)	0.128
2. P-tau	−0.154 (−0.394, 0.087)	0.207	−0.088 (−0.323, 0.147)	0.455
3. T-tau	−0.032 (−0.057, −0.006)	0.016	−0.022 (−0.048, 0.004)	0.095

a Unstandardized coefficient.

b SCD/MCI vs dementia. Mini-BESTest, Mini-Balance Evaluation Systems Test; TUG, Timed Up-and-Go; TUG DTC, Timed Up-and-Go dual-task cost.

In the sensitivity analyses with the subsample that had MCI or dementia due to AD (n = 54), Aβ_42_ was associated with Mini-BESTest (0.007 [0.002, 0.011], *p* = 0.003) and TUG (−0.004 [−0.007, −0.001], *p* = 0.021), but not with TUG DTC nor with gait speed in the adjusted regression models. P-tau_181_ and T-tau were not significantly associated with any of the mobility outcomes in this subsample.

## 4. Discussion

The main findings from this study of memory clinic patients were that while Aβ_42_ was associated with several aspects of mobility, tau-pathology was not associated with any of the mobility outcomes after adjusting for diagnostic status.

The strongest correlations in our study were observed between the Mini-BESTest, representing dynamic balance control, and levels of Aβ_42._ This association was also significant in the subsample with AD pathology, as well as in the adjusted models. While balance is closely associated with degree of cognitive impairment (Tangen et al., 2014; Yan et al., 2022), few studies have examined its relationship with degree of neurodegeneration. In Nilsson et al.’s study, longer time to walk the figure-of-eight test (requiring dynamic balance during walking) was associated with pathological Aβ_42_/Aβ_40_ in memory clinic patients with SCD and MCI, thus their results are in line with ours (Nilsson et al., 2021). However, in their study, this association was not statistically significant in the SCD or MCI groups separately. Further support for the association between balance and amyloid pathology can be seen through studies of falls, where a very large proportion of cognitively normal hip fracture patients had abnormal Aβ_42_/Aβ_40,_ P-tau, and T-tau levels (Oh et al., 2018), and Aβ_42_ was associated with shorter time to first fall in older adults with preclinical AD (Stark et al., 2013).

Gait and TUG has been studied in relation to amyloid and tau in several previous studies, with conflicting results. In our study, higher gait speed was correlated with higher levels of Aβ_42_, but this association was not significant in the adjusted models. This is overall in line with two studies of memory clinic patients where Muurling et al. found no association between gait and Aβ_42_ levels (Muurling et al., 2020), while Koychev and colleagues reported that gait was associated with the Aβ_42_/Aβ_40_ and Aβ_42_/Aβ_38_ ratios, but not with Aβ_42_ (Koychev et al., 2018). In our study, the lack of association could be explained by gait speed being a too simple outcome to detect subtle changes. However, this is not the case in the studies by Muurling et al. (2020) and Koychev et al. (2018), where they used sophisticated instrumented gait assessments deriving data on qualitative aspects of gait. The TUG test represents more than gait alone, as in addition to walking it also involves rising up, turning, and sitting down. This complexity might explain why in contrast to gait speed, TUG was significantly associated with Aβ_42_ in all our analyses. However, none of the other studies found significant associations between TUG and amyloid in memory clinic patients (Nielsen et al., 2018; Ahman et al., 2019; Muurling et al., 2020; Nilsson et al., 2021).

Dual-task gait cost is suggested as brain stress test to detect mobility problems in older adults, as it combines walking with a cognitive attention-demanding task (Cullen et al., 2018). However, in our study, the associations between TUG DTC and Aβ_42_ and T-tau were no longer significant when we also adjusted for diagnostic status. Overall, these results are in line with findings from Nilsson et al. (2021) and Nielsen et al. (2018) while Ahman et al. (2019) did not find any association between biomarkers and increased dual-task gait cost. Interestingly, in our study, the associations between TUG DTC, Aβ_42_ and T-Tau were not significant in the subgroup analyses, which may indicate that the association was driven by participants with non-AD pathology.

The only significant association between tau and the different mobility outcomes in our study was between T-tau and TUG DTC. Dual-task gait cost was also associated with tau in the other studies (Nielsen et al., 2018; Muurling et al., 2020; Nilsson et al., 2021) except from Åhman’s study where only the cognitive cost of the dual task was associated with tau (Ahman et al., 2019).

A potential source for some of the heterogeneous findings across these studies could be inclusion of different samples regarding age or degree of cognitive impairment. In our study, all participants were recruited from a memory clinic and most had mild cognitive dysfunction, also the patients with dementia. Thus, we have a sample with more severe cognitive impairment than for example Nilsson who did not include patients with dementia (Nilsson et al., 2021) or Muurling et al. (2020) and Nielsen et al. (2018) who also included cognitively healthy older adults in addition to patients with MCI and dementia. Åhman’s study included participants comparable to the present study, including memory clinic patients with SCD, MCI, and dementia, but their sample’s MMSE score (median score 25 points) was lower than in our study (median score 27 points; Ahman et al., 2019). Age is also important as pathological levels of Aβ_42_ and tau becomes more prevalent with age (Jack et al., 2017). The different cohorts in the mentioned studies appear to be of similar age as ours, with Nilsson’s sample being the oldest with a mean age of 71.8 years and Koychev’s sample the youngest with mean age of 67 years (Koychev et al., 2018; Nilsson et al., 2021).

Dual-task gait requires attentional resources and executive functions (Hobert et al., 2011) and dual-task gait assessment is reported to increase the sensitivity of gait analysis to differentiate between MCI and cognitively healthy older adults (Bahureksa et al., 2017). In our study, the associations between mobility and levels of Aβ_42_ are supported by the significantly worse performance by the dementia group compared to the combined SCD/MCI group on both TUG DTC and Mini-BESTest. Less well-explored than gait and dual task, Mini-BESTest was the mobility outcome with the strongest correlation with levels of Aβ_42_. Dynamic balance may emerge as a motor skill closely interrelated with cognitive function, and more studies should explore this association at an earlier stage of the AD continuum. Still, it is important to keep in mind that the correlation between mobility and levels of Aβ_42_ was rather weak if one aims to consider mobility as a surrogate marker for ongoing dementia pathology.

### 4.1. Strengths and limitations

A limitation of our study is the rather small sample size. Still, our cohort is representative of those patients who have lumbar puncture done as part of their diagnostic process based on clinical indication. Thus, we cannot generalize our results to persons with moderate to severe level of dementia, nor to the oldest-old. Also, since we did not have data on the Aβ_42_/Aβ_40_ and Aβ_42_/Aβ_38_ ratios, there are limitations to how comparable our results are to the findings from studies using these ratios (Koychev et al., 2018; Nilsson et al., 2021).

Another limitation of the study is that gait speed was done on two different lengths (4 vs. 6 m), in the two cohorts. The gait speed was significantly higher in the cohort where the m/s was derived from the 6 m, probably representing a longer phase after the acceleration phase. This can be an explanation for the relatively weak association between gait speed and biomarkers in the present study. An important strength of the study is the standardization of procedures. The lumbar punctures followed standard procedures and the CSF biomarker analyses were all carried out at the same laboratory. All the mobility assessments were carried out by the same experienced physiotherapist. Also, the Mini-BESTest is likely the most comprehensive assessment of balance done in relation to the AD core biomarkers, providing new knowledge to the field.

In conclusion, we found that better results on the performance-based mobility outcomes were associated with higher levels of CSF Aβ_42,_ but not with tau-pathology. The association was particularly strong between Aβ_42_ and Mini-BESTest, suggesting that dynamic balance might be closely interrelated with AD-specific pathology. Longitudinal studies are needed to shed light on how changes in mobility and AD pathology are related, and these associations should also be explored in primary care settings.

## Data availability statement

The original contributions presented in the study are included in the article/supplementary material, further inquiries can be directed to the corresponding author.

## Ethics statement

The studies involving human participants were reviewed and approved by the Regional Committee for Medical and Health Research Ethics South East Norway. The patients/participants provided their written informed consent to participate in this study.

## Author contributions

GT, KS, KT, and A-BK designed the study. GT analyzed the data and drafted the manuscript. All authors contributed to the article and approved the submitted version.

## Funding

The Southern and Eastern Norway Regional Health Authority supported the study through funding for GT (grant number 2016099).

## Conflict of interest

A-BK, KP, and PB-W report work with Roche drug trial BN29553 and Novo Nordisk drug trial NN6535-4730 trial. A-BK report work with Boehringer-Ingelheim drug trial 1346.0023.

The remaining authors declare that the research was conducted in the absence of any commercial or financial relationships that could be construed as a potential conflict of interest.

## Publisher’s note

All claims expressed in this article are solely those of the authors and do not necessarily represent those of their affiliated organizations, or those of the publisher, the editors and the reviewers. Any product that may be evaluated in this article, or claim that may be made by its manufacturer, is not guaranteed or endorsed by the publisher.
